# Research progress on mechanisms of tumor immune microenvironment and gastrointestinal resistance to immunotherapy: mini review

**DOI:** 10.3389/fimmu.2025.1641518

**Published:** 2025-07-25

**Authors:** Zheng Zhang, Yangping Wu

**Affiliations:** ^1^ Division of Liver Surgery, Department of General Surgery and Laboratory of Liver Surgery, West China Hospital, Sichuan University, Chengdu, Sichuan, China; ^2^ Department of Pulmonary and Critical Care Medicine, Targeted Tracer Research and Development Laboratory, Institute of Respiratory Health, Frontiers Science Center for Disease-related Molecular Network, Precision Medicine Key Laboratory of Sichuan Province and Precision Medicine Research Center, West China Hospital, Sichuan University, Chengdu, Sichuan, China

**Keywords:** gastrointestinal tumors, tumor immune microenvironment, immunotherapy resistance, immune checkpoint inhibitors, combination therapy, mRNA vaccines

## Abstract

Gastrointestinal (GI) tumors present a major clinical challenge due to complex immune evasion mechanisms and generally poor responses to immunotherapy. Tumor immune microenvironment (TIME) is a critical determinant of immunotherapy resistance. Immunosuppressive cell populations such as regulatory T cells, M2-polarized tumor-associated macrophages, and cancer-associated fibroblasts, together with aberrant cytokine networks and mechanical stress in the tumor stroma, cooperate to exclude T-cell infiltration and induce T-cell exhaustion, thereby undermining the efficacy of immune checkpoint inhibitors. In addition, TIME-driven signaling pathway activation and epigenetic reprogramming further reinforce immune escape and therapeutic failure. Recent advances in single-cell and spatial transcriptomic technologies have greatly improved our understanding of these processes. Meanwhile, strategies including multi-target combination immunotherapies, epigenetic modulators, mRNA vaccines, and gut microbiota interventions are under active investigation to reverse resistance and remodel the TIME. This mini review summarizes the multifaceted mechanisms of immunotherapy resistance in GI tumors and discusses the potential of emerging therapeutic strategies to improve clinical outcomes.

## 1 Introduction

Gastrointestinal malignancies (including gastric, colorectal, and gastrointestinal stromal cancers) remain among the leading causes of cancer incidence and mortality worldwide (1). Despite significant progress in immunotherapy for GI tumors, the issue of therapeutic resistance continues to pose a major challenge in the clinic. Currently, immune checkpoint inhibitors (ICIs) such as anti–PD-1/PD-L1 antibodies have shown robust efficacy in a subset of GI tumors with high microsatellite instability (MSI-H) or deficient mismatch repair (dMMR) (2). However, for the majority of patients with microsatellite stable (MSS) or proficient mismatch repair (pMMR) tumors, single-agent immunotherapy yields poor responses, characterized by varying degrees of resistance, low response rates, and limited survival benefits (3). The multidimensional nature of resistance (including genetic mutations, epigenetic regulation, and tumor microenvironment remodeling) further exacerbates the difficulty of clinical management.

The growing evidence shows that GI tumor immunotherapy resistance is closely related to the tumor immune microenvironment (TIME) (4). The TIME is a complex ecosystem composed of diverse cellular and acellular components; resistance mechanisms driven by the TIME are essentially mediated by the dynamic crosstalk between heterogeneous cancer cells and stromal elements, orchestrated through multi-level regulatory networks (5). In GI tumors, heterogeneous cancer cells secrete various cytokines and chemokines, forming a complex network of immunosuppressive signals. Cancer cells and immunosuppressive cells (such as tumor-associated macrophages and myeloid-derived suppressor cells) produce factors, such as IL-10 and TGF-β, (6) and express checkpoint ligands (e.g. PD-L1) that inhibit T-cell activation and proliferation, leading to T-cell dysfunction and exhaustion and reducing their cytotoxicity (7). M2-polarized TAMs secrete IL-10, VEGF and other factors promote angiogenesis and immunosuppression, thereby dampening anti-tumor activity of T cells (8). Cancer-associated fibroblasts (CAFs) in the TIME secrete cytokines and remodel the extracellular matrix to create a physical barrier that hinders immune cell infiltration, further promoting immunosuppression and tumor progression (9).

In recent years, the application of novel therapies such as mRNA vaccines, oncolytic viruses, fecal microbiota transplantation, and combinations of immunotherapy with chemotherapy/radiotherapy, or targeted inhibitors, as well as deeper investigation into microenvironment driven resistance mechanisms have provided encouraging evidence in preclinical and clinical studies. In summary, this review will detail the latest findings on how various immune and stromal components of the TIME contribute to immunotherapy resistance in GI tumors and will highlight emerging therapeutic strategies aimed at overcoming resistance and improving clinical outcomes.

## 2 Mechanisms of immunotherapy resistance in the tumor immune microenvironment

The tumor immune microenvironment (TIME) encompasses all immune-related cells and factors in the vicinity of tumor cells and plays a pivotal role in tumor development, progression, metastasis, and therapy response. The immune microenvironment of GI tumors is highly complex, comprising immune cells, non-immune stromal cells, cytokines, chemokines, and extracellular matrix components (10). A variety of immunosuppressive cells—including regulatory T cells (Tregs), myeloid-derived suppressor cells (MDSCs), and M2-polarized TAMs—accumulate in the TIME (11). These cells secrete immunosuppressive cytokines (e.g. IL-10, TGF-β) and express inhibitory checkpoints (e.g. PD-1/PD-L1 and CTLA-4) which suppress effector T-cell function and facilitate immune escape (12). Non-immune stromal elements in the TIME (including CAFs, endothelial cells, and others) further contribute by secreting factors and restructuring the extracellular matrix to create physical barriers against immune cell infiltration. In addition to cellular components, soluble cytokines and chemokines in the TIME (e.g. IL-10, TGF-β, CXCL12) actively regulate immune cell function and migration (13). Multiple studies have demonstrated that enrichment of immunosuppressive cells together with abundant suppressive factors and checkpoint ligands in the TIME promotes immune evasion, rendering single-agent checkpoint blockade largely ineffective (14). With advances in single cell sequencing and spatial transcriptomics, our understanding of the heterogeneity and functional organization of the TIME has greatly deepened (15–17). All of these insights are paving the way for new diagnostic and therapeutic approaches.

### 2.1 T-cell infiltration and exhaustion

The degree of T-cell tumor infiltration and the functional state of those T cells—especially the phenomenon of T cell exhaustion—have a direct impact on immunotherapy efficacy. In GI tumors, the density of tumor infiltrating T cells correlates strongly with patient prognosis (18). Clinically, high levels of CD3^+^ T cell infiltration are associated with improved overall survival, suggesting that tumors may evade immune surveillance via adaptive immune resistance mechanisms (19). In GI cancers, sustained exposure of CD8^+^ T cells to tumor-associated antigens or neoantigens can drive them into a state of functional exhaustion characterized by upregulation of multiple inhibitory receptors and diminished cytokine production and cytotoxicity (20). For example, exhausted CD8^+^ T cells in the tumor express high levels of PD-1 and TIM-3, and show reduced effector function (21). Jin et al. (22) reported that in gastric signet-ring cell carcinoma, high infiltration of CD3^+^ T cells was linked to better survival, although many of these T cells expressed PD-1, indicating ongoing adaptive immune resistance (22). More recently, Ding et al. (23) identified that CAFs secreting IL-8 can upregulate PD-1 expression on CD8^+^ T cells, thereby promoting T-cell exhaustion in the gastric cancer microenvironment (23). In addition, in gastric cancer, Duan et al. (24) demonstrated that CD39 marked a subset of tumor-infiltrating CD4^+^ T cells that exhibited an exhausted, immunosuppressive effector-memory phenotype, while, inhibiting CD39 enzymatic activity reinvigorated these CD4^+^ T cells, thereby enhancing their secretion of TNF-α and IFN-γ (24). Together, these findings underscore that insufficient T-cell infiltration and the presence of exhausted T-cell populations limit the effectiveness of ICIs in most GI tumors.

### 2.2 Macrophage infiltration and M2 polarization

Tumor-associated macrophages (TAMs) play an essential role in tumorigenesis, progression, metastasis, and immune evasion, with their abundance and polarization profoundly influencing immune escape mechanisms (25). M2-polarized TAMs, in particular, are central drivers of immunotherapy resistance in GI tumors, fostering an immunosuppressive milieu that blunts treatment efficacy (26). TAMs originate from two main sources: tissue-resident macrophages and bone marrow derived circulating monocytes that infiltrate the tumor and differentiate in situ (27, 28). Tumor cells secrete chemoattractants such as CCL2 and VEGF to recruit monocytes from the blood into the tumor microenvironment. Once cytokines enter the tumor, like TGF-β, IL-4, and IL-10 they could drive the differentiation and polarization of these macrophages toward an M2-like phenotype via activation of pathways including JAK/STAT, PI3K/Akt, and NF-κB (29–31). For example, Laviron and colleagues (32) have shown that tissue-resident macrophages and monocyte-derived macrophages can play distinct roles in tumors, but under the influence of tumor-derived factors, both can be co-opted into pro-tumoral TAMs (32).

In the context of immunotherapy resistance, M2 TAMs contribute through multiple mechanisms. First, M2 TAMs secrete immunosuppressive cytokines (e.g. IL-10, TGF-β) that directly impair the function of effector T cells, weakening anti-tumor immune responses and promoting tumor immune escape (33). Second, M2 TAMs overexpress immune checkpoint ligands such as PD-L1 in the tumor microenvironment; engagement of these ligands with PD-1 on T cells transmits inhibitory signals that further diminish T-cell activity (34). In gastric cancer, M2 TAM–derived exosomes carrying microRNA-21 (miR-21) have been shown to regulate the APOE/PTEN/PI3K/Akt pathway in tumor cells, thereby enhancing resistance to cisplatin chemotherapy (35). He et al. (36) reported that gastric cancer cells with high YAP1 expression secrete IL-3, which skews macrophages toward an M2 phenotype and induces GLUT3-dependent metabolic reprogramming, thereby promoting 5-FU resistance (36). In colorectal cancer, a novel mechanism of cetuximab resistance involves a long non-coding RNA (LncRNA HCG18) in tumor cells that influences the miR-365a-3p/FoxO1/CSF-1 axis, leading to enhanced M2 polarization of TAMs and subsequent therapeutic resistance (37). Additionally, M2 TAMs can secrete factors like pleiotrophin (PTN) that interact with receptors on cancer stem cells, endowing tumor cells with stem-like properties and further increasing resistance and metastatic potential (38). Collectively, these mechanisms illustrate how TAM enrichment and M2 polarization in the TIME not only facilitate immune evasion but also contribute to treatment failure with both ICIs and conventional therapies.

### 2.3 Cancer associated fibroblast differentiation and activity

Cancer-associated fibroblasts (CAFs) are the predominant stromal cell type in the tumor microenvironment of GI cancers and have high functional heterogeneity as revealed by single-cell sequencing (39). CAFs play crucial roles in tumor growth, progression, and resistance. Studies have shown that CAFs can arise from multiple cell types in the tumor microenvironment, including resident tissue fibroblasts, adipocytes, pericytes, stellate cells, mesothelial cells, pericrypt fibroblasts, and even mesenchymal stem cells (40, 41). In GI tumors, the precise origins of CAFs remain incompletely defined, but evidence from mouse models indicates that a significant fraction of CAFs can derive from bone marrow–mesenchymal stem cells (MSCs) (42). For instance, about 20% of CAFs in a mouse model of gastric cancer were shown to originate from bone marrow–derived MSCs, a finding corroborated in patients who underwent bone marrow transplantation and later developed gastric or rectal cancer (43). In liver cancer, hepatic stellate cells are a likely source of CAFs (44), whereas in colitis-associated colorectal cancer, Lepr^+^ stromal cells have been observed to proliferate and convert into CAFs expressing the marker CD146 (40, 45). Pancreatic stellate cells, upon activation, are known to transform into αSMA^+^ CAFs in pancreatic cancer (46). These observations underscore the diverse provenance of CAF populations across different GI tumors.

The differentiation and activation of CAFs are driven by signals from tumor cells and the microenvironment. TGF-β is considered a key inducer of myofibroblastic CAF (myCAF) differentiation. Tumor cell secreted TGF-β can convert local fibroblasts into pro-tumorigenic myCAFs (47). Crosstalk between fibroblasts and cancer cells via NF-κB activation and IL-6/IL-8 secretion further supports CAF activation (48). The Notch signaling pathway has also been implicated in CAF differentiation (49). In addition to specific pathways, broader factors such as exposure to tumor cell conditioned media, hypoxic conditions, and cancer-derived exosomes can trigger normal stromal cells to acquire a CAF phenotype (50, 51). For example, hypoxia and tumor exosomes have been shown to induce normal fibroblasts to express CAF markers and functions (52).

CAFs contribute to immunotherapy resistance through multiple avenues. Arpinati and Scherz-Shouval (53) demonstrated that CAFs in GI tumors secrete immunosuppressive factors (TGF-β, IL-6, etc.) that inhibit T-cell activation and infiltration while recruiting Tregs and MDSCs, thereby creating an immune-excluded microenvironment that diminishes immunotherapy efficacy (53, 54). CAFs can also release prostaglandin E2 (PGE2), which suppresses natural killer (NK) cell activity and weakens anti-tumor immune responses (55). Moreover, CAFs upregulate the expression of immune checkpoint molecules (e.g. PD-L1, B7-H3, IDO) on themselves or nearby cells, directly impairing T-cell function and facilitating immune escape (56). Zhong et al. (57) found that CAF-secreted cytokines like IL-6 and IL-8 sustain cancer stem cell survival and proliferation, thereby increasing tumor tolerance to immunotherapy (57). CAF-derived signals (such as through TGF-β) can also maintain cancer stemness and enhance resistance to therapies (58). Importantly, CAFs remodel the tumor extracellular matrix (ECM) by producing and cross-linking collagen and other matrix components, which not only acts as a physical barrier limiting drug and immune cell penetration, but also creates a protective niche for tumor cells (59). This dense, fibrotic stroma characteristic of many GI tumors (e.g. pancreatic and scirrhous gastric cancers) has been associated with poor immune infiltration and resistance to both chemotherapy and ICIs. Indeed, Akiyama et al. (60) showed that highly fibrotic tumors respond poorly to anti PD-1 therapy, and that dual inhibition of PDGFRα/β on stromal cells can “reprogram” the stroma to be less fibrotic and more permissive to T-cell infiltration, thereby enhancing ICI efficacy (60). Taken together, these findings illustrate that CAFs are key architects of an immunosuppressive, therapy-resistant tumor microenvironment in GI cancers.

### 2.4 Megakaryocytes/platelets and immune response

Megakaryocytes (MKs) are the largest hematopoietic cells, primarily responsible for producing platelets and known to participate in hemostasis and immune regulation. Recent studies suggest that MKs play important roles in the development and progression of GI tumors, especially gastric cancer, by modulating the immune microenvironment and promoting pro-thrombotic conditions (61–63). High platelet counts and a hypercoagulable state are correlated with poor prognosis in many malignancies, including gastric cancer. GI tumor cells can release metabolites such as kynurenine, which activate the aryl hydrocarbon receptor (AhR)–RUNX1 axis in hematopoietic progenitors, skewing their differentiation toward megakaryocytes at the expense of erythroid lineages and thereby leading to thrombocytosis and tumor-associated hypercoagulability (64). In gastric cancer, *Fusobacterium nucleatum* infection and other factors can promote deep vein thrombosis, partly through the induction of neutrophil extracellular traps (NETs) which stimulate thrombogenesis. NETs can release extracellular vesicles rich in proteins like 14-3-3ϵ that are taken up by hematopoietic progenitors to activate the PI3K/Akt pathway, driving their differentiation into MKs and causing elevated platelet counts (65). This hypercoagulable, pro-thrombotic environment not only increases the risk of thromboembolic events but also contributes to an immunosuppressive niche; platelets can shield circulating tumor cells and modulate immune cell trafficking and function.

Furthermore, metastatic colorectal cancer patients showed upregulation of *Erbin* in platelets/MKs. This was found to suppress B-cell–mediated anti-tumor immunity via a metabolic mechanism: loss of Erbin in MKs boosted their mitochondrial oxidative phosphorylation and production of acylcarnitines delivered to B cells, which in turn enhanced B cell metabolic fitness and promoted anti-tumor T-cell responses by facilitating PD-1 degradation on T cells. Knockout of *Erbin* improved anti-tumor immunity in preclinical models, highlighting platelets/MKs as potential targets to modulate the immune niche (66). Collectively, these findings reveal that MKs and platelets significantly influence the TIME and, thus, patient responses to immunotherapy. Thrombocytosis and platelet activation often accompany cancer progression and are associated with immune suppression. Targeting the interplay between coagulation, platelets, and immune cells may therefore represent a novel approach to enhance immunotherapy efficacy in GI cancers.

### 2.5 Biophysical stress and immune resistance

Biophysical properties of the tumor and its microenvironment such as mechanical stress, tissue stiffness, cellular morphology, and shear forces can directly impact immune cell recognition and killing of tumor cells, thereby influencing resistance to immune therapies (67, 68). Tumor cells in GI cancers often experience and adapt to mechanical stresses (solid stress from proliferating cells and desmoplastic stroma, fluid shear stress in circulation, etc.), which can in turn induce cellular programs that promote immune evasion and drug resistance (69–71). For instance, mechanical forces have been shown to drive epithelial mesenchymal transition (EMT) and autophagy in tumor cells, which are linked to immune escape and resistance (72, 73). Noman et al. (74) demonstrated that solid stress in tumors can compress blood vessels, leading to hypoxia and acidosis, which impair immune cell infiltration and function. Solid stress can also induce EMT in cancer cells, making them more resistant to cytotoxic T lymphocyte (CTL) killing via upregulation of anti-apoptotic pathways and immune checkpoints like PD-L1 (74). In fact, increased EMT has been correlated with higher PD-L1 expression and greater recruitment of Tregs and MDSCs, contributing to immunosuppression (75). Onwudiwe et al. (76) found that reducing tissue stiffness or breaking down fibrosis in pancreatic tumors improved T-cell infiltration and sensitivity to ICIs, underscoring the link between biomechanical factors and immune exclusion (76).

Fluid shear stress is another important mechanical factor, especially relevant for circulating tumor cells and metastatic spread (77). When tumor cells enter the bloodstream, they are exposed to shear forces in the range of 1–30 dyn/cm^2^ (78). Exposure to high shear stress can induce changes in tumor cell morphology (e.g. from polygonal to spindle shape) by downregulating E-cadherin and upregulating N-cadherin and β-catenin, effectively promoting EMT and enhancing migratory capacity (79). Shear stress has also been shown to activate autophagy in circulating tumor cells as a protective mechanism against mechanical damage. Yu et al. (80) reported that fluid shear stress can upregulate immunosuppressive molecules via mechanotransduction pathways (such as YAP/TAZ signaling), thereby helping tumor cells evade immune detection under flow conditions (80).

Moreover, abnormal tumor vasculature contributes to irregular interstitial fluid flow and areas of high interstitial pressure. VEGF, a key angiogenic factor often overexpressed in GI tumor microenvironments, directly suppresses CTL function and inhibits dendritic cell maturation and antigen presentation, which hampers T-cell activation (81). VEGF also recruits immunosuppressive cells (Tregs, MDSCs, M2 TAMs) to tumors (82). The net effect is a vicious cycle where mechanical abnormalities in the TME (driven by factors like VEGF) lead to hypoxia and low pH, which further promote immunosuppression and resistance (83). Indeed, alleviating tumor hypoxia (e.g. by normalizing vasculature or reducing solid stress) has been shown to improve responses to immunotherapy in some models (84).

In summary, the physical and mechanical characteristics of tumors in GI cancers significantly affect immune cell behavior and therapy response. Strategies to modulate these biophysical factors such as drugs to reduce desmoplasia and solid stress, normalize vasculature, or disrupt shear-induced survival signals could enhance immune infiltration and restore sensitivity to immunotherapies.

**Figure 1:**
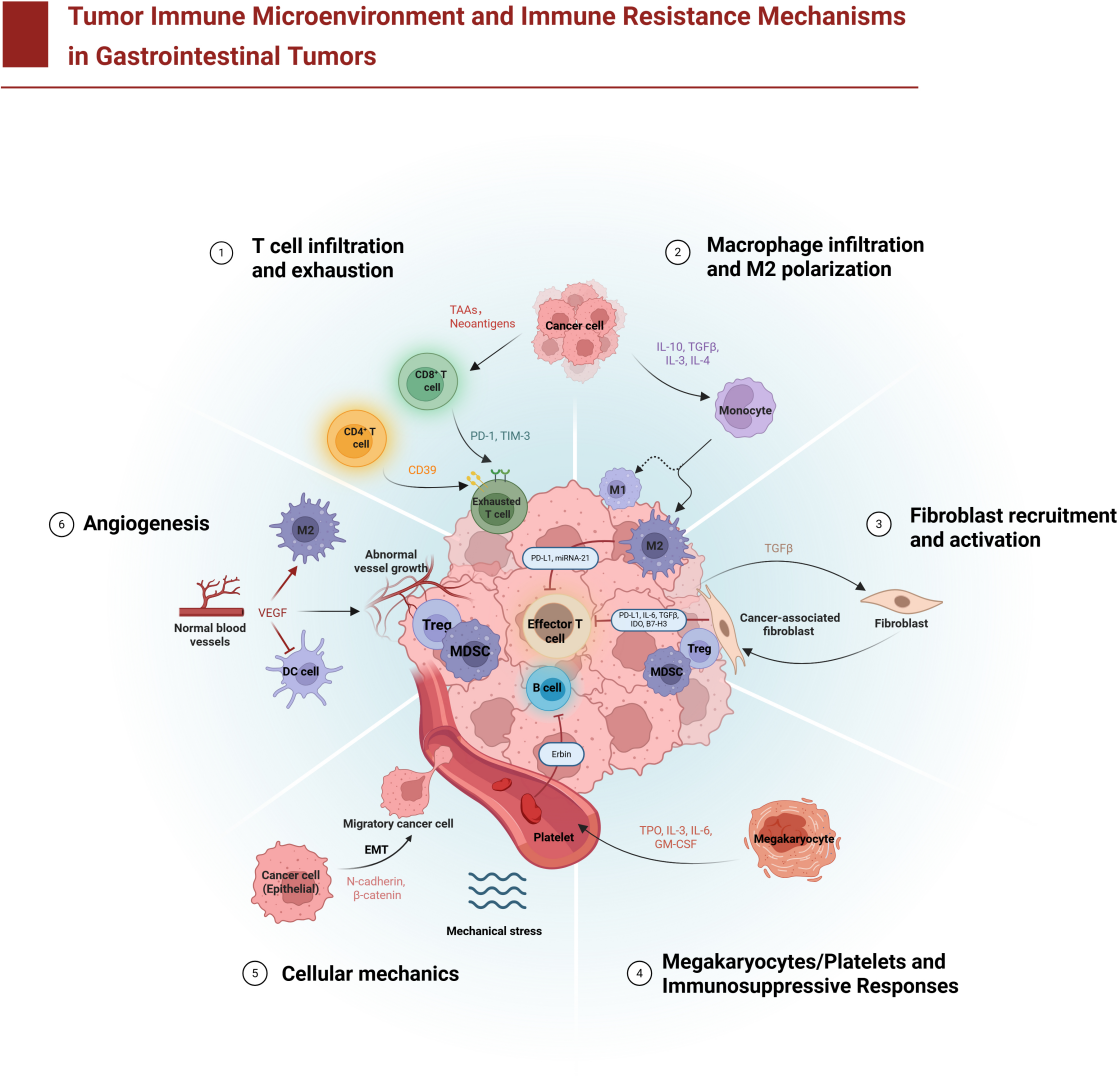
Tumor immune microenvironment and immune resistance mechanisms in gastrointestinal tumors. The diagram illustrates six major mechanisms by which gastrointestinal tumors evade immune attack: (1) T cell infiltration and exhaustion **–** CD8^+^ cytotoxic T lymphocytes (T cells) infiltrate the tumor but become functionally exhausted due to chronic antigen exposure and inhibitory checkpoint signals (e.g., programmed cell death protein 1 (PD-1), T-cell immunoglobulin and mucin-domain 3 (TIM-3), and the ectoenzyme CD39) delivered by tumor cells and immunosuppressive cells like regulatory T cells (Tregs) and myeloid-derived suppressor cells (MDSCs); (2) Macrophage infiltration and M2 polarization **–** monocytes are recruited to the tumor and differentiate into tumor-associated macrophages (TAMs) that adopt an anti-inflammatory M2 phenotype under the influence of tumor-derived factors (e.g., interleukin-6 (IL-6), transforming growth factor-beta (TGF-β)), leading TAMs to secrete immunosuppressive cytokines and promote tumor growth; (3) Fibroblast recruitment and activation **–** cancer-associated fibroblasts (CAFs) are activated and accumulate in the stroma, where they secrete TGF-β and deposit abundant extracellular matrix (ECM), increasing ECM stiffness and forming a physical barrier that impedes immune cell penetration; (4) Megakaryocyte/platelet-mediated immunosuppression **–** megakaryocyte-derived platelets aggregate with tumor cells and release immunosuppressive mediators (such as TGF-β), effectively cloaking tumor cells from immune recognition and facilitating metastatic spread; (5) Tumor cell mechanical adaptation **–** tumor cells undergo epithelial–mesenchymal transition (EMT) and adapt to mechanical stresses (such as fluid shear stress and high interstitial pressure), enhancing their invasiveness and survival while resisting immune cell–mediated killing; and (6) Abnormal angiogenesis **–** excessive vascular endothelial growth factor (VEGF) and other pro-angiogenic signals drive the formation of abnormal, leaky blood vessels, creating a hypoxic microenvironment that hinders effective immune cell infiltration. These diverse cellular components (T cells, Tregs, B lymphocytes (B cells), MDSCs, TAMs, CAFs, and platelets) and their molecular mediators collectively establish an immunosuppressive tumor microenvironment that allows gastrointestinal cancers to resist immune surveillance.

**Figure 1**: Schematic overview of how components of the TIME contribute to immunotherapy resistance in GI tumors.

## 3 Immunotherapeutic strategies to overcome resistance

**Figure 2:**
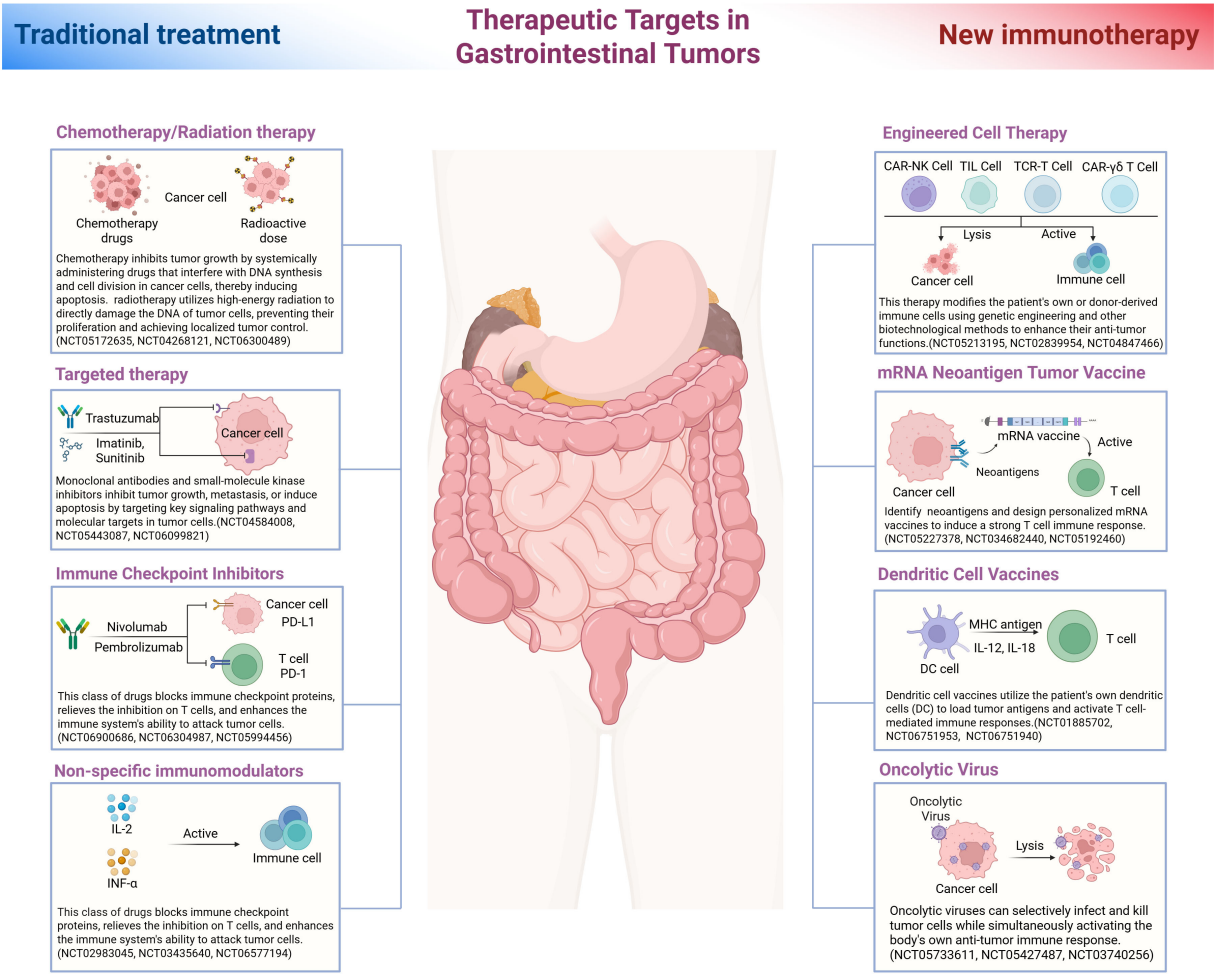
Therapeutic targets in gastrointestinal tumors. This illustration compares traditional treatment strategies with novel immunotherapeutic approaches for gastrointestinal (GI) cancers, arranged on the left and right sides, respectively, around a central depiction of the digestive tract. The left side shows conventional therapies including chemotherapy and radiotherapy, which directly damage tumor cell DNA and induce cancer cell death. Targeted therapies are also depicted, exemplified by trastuzumab (a monoclonal antibody against human epidermal growth factor receptor 2 (HER2)) and imatinib (a tyrosine kinase inhibitor used in KIT-mutant gastrointestinal stromal tumors (GISTs)), both of which inhibit specific oncogenic pathways. Immune checkpoint inhibitors, such as nivolumab and pembrolizumab (anti–PD-1 antibodies), block inhibitory signals on T cells and thereby enhance anti-tumor immunity. Non-specific immunomodulators, such as interleukin-2 (IL-2) and interferon-α (IFN-α), are also depicted; these cytokines broadly stimulate the immune response. On the right side, emerging immunotherapeutic modalities include engineered cell therapies such as CAR T cells (chimeric antigen receptor T cells), CAR-engineered natural killer (NK) cells, and adoptive transfer of tumor-infiltrating lymphocytes (TILs), which employ *ex vivo* expanded or genetically engineered immune cells to recognize and kill cancer cells. Vaccination strategies are also depicted, including personalized neoantigen vaccines (for example, mRNA vaccines encoding tumor-specific antigens) and dendritic cell vaccines that prime the immune system by presenting tumor antigens to T cells. Additionally, oncolytic viruses are illustrated; these selectively infect and lyse tumor cells while stimulating anti-tumor immune responses. Arrows indicate each therapy’s cellular target (tumor cells or immune cells) in the diagram. Each approach is labeled with a representative clinical trial identification number (NCT ID) indicating ongoing clinical evaluation.

Given the multifactorial nature of resistance in the GI tumor immune microenvironment, a variety of strategies are being explored to counteract these mechanisms and improve patient responses. Combination approaches that target multiple pathways in the TIME have shown promise. Here we discuss emerging therapeutic strategies, including combination therapies, cancer vaccines, microbiome modulation, and advanced cell therapies, which aim to reprogram the TIME and overcome immunotherapy resistance. Several of these strategies are currently under preclinical and clinical investigation (**Figure 2**).

### 3.1 Combination therapies

Chemo-immunotherapy: Combining traditional chemotherapy with immunotherapy is a rational strategy, as certain chemotherapeutic agents can modulate the TIME to be more immunogenic. Chemotherapy can induce immunogenic cell death of tumor cells, increase tumor mutational burden (and thus neoantigen availability), and deplete immunosuppressive cell populations like Tregs and MDSCs, collectively enhancing the efficacy of ICIs. In a phase II clinical study, a neoadjuvant regimen of anti PD-L1 (atezolizumab) “induction” followed by chemotherapy in respectable gastric/gastroesophageal junction adenocarcinoma was found to be safe and feasible, achieving a major pathological response rate (~70% MPR) significantly higher than historical controls (with 45% pathologic complete response in that study) (85). These results suggest that upfront ICI therapy may prime the immune system, and subsequent chemotherapy can further expose tumor antigens and reduce suppressive cells, achieving synergistic effects.

Radio-immunotherapy: Local radiotherapy has immunomodulatory effects such as releasing tumor antigens, inducing immunogenic cell death, and activating type I interferon pathways (86). These effects can provoke a systemic anti-tumor immune response even at unirradiated sites (the “abscopal effect”). Radiotherapy has been shown to significantly increase response rates to ICIs in patients who initially did not respond to checkpoint blockade (87). Liu et al. (88) reported early results that radiotherapy combined with anti PD-1 therapy led to increased T-cell activation and suppression of immunosuppressive pathways, enhancing anti-tumor efficacy in GI- tumors (88). Clinical trials are ongoing to determine the optimal timing and dosing of radiotherapy to maximize this synergy with ICIs (NCT04535024, NCT02608385). Furthermore, targeting the desmoplastic stroma can also augment immunotherapy: for example, the anti-fibrotic drug pirfenidone was found to reduce CAF activity in gastric cancer, thereby increasing tumor sensitivity to both chemotherapy and PD-1 blockade (89).

Multi-checkpoint blockade: Tumor cells often develop resistance to single-agent PD-1/PD-L1 inhibitors by upregulating alternative inhibitory receptors such as LAG-3, TIGIT, and TIM-3 (90). These compensatory pathways are key drivers of acquired resistance to immunotherapy. Consequently, dual or multi-checkpoint blockade strategies are being pursued. Co-blockade of LAG-3 and PD-1 has demonstrated enhanced anti-tumor immune responses in preclinical and clinical settings. In a clinical trial of advanced esophageal cancer treated with pembrolizumab (anti PD-1) as ≥ second-line therapy, gene expression profiling revealed co-enrichment of LAG3 and IDO1 in PD-L1^+^ tumors, and high LAG-3 expression was associated with poorer progression-free survival (91). These findings support LAG-3 as a rational co-target. TIGIT is another checkpoint of interest; TIGIT suppresses CD8^+^ T and NK cell activation and represents an important compensatory pathway in PD-1 resistant tumors (92). In MSS colorectal cancer models and early trials, combined TIGIT and PD-1 blockades have shown potential to reinvigorate exhausted CD8^+^ T cells, reduce tumor burden, and possibly prolong survival (93). TIM-3 is often co-expressed with PD-1 on T cells across multiple solid tumors; its upregulation is closely associated with anti PD-1 failure (94). In colorectal and gastric cancers, high TIM-3 expression in tumor tissues or peripheral blood correlates with worse overall and progression-free survival, marking it as a biomarker of resistance and poor prognosis (95). Trials combining anti TIM-3 with anti PD-1 are being explored.

Epigenetic therapy plus immunotherapy: The immunosuppressive TIME is partly maintained by epigenetic programming in both tumor and immune cells. Therefore, using epigenetic modulators can potentially “reset” these programs. DNA methyltransferase inhibitors (DNMTis) and histone deacetylase inhibitors (HDACis) can upregulate antigen presentation machinery, increase tumor-infiltrating lymphocytes, and stimulate type I IFN signaling, converting “cold” tumors into “hot” ones more susceptible to ICIs (96). Early-phase clinical trials in colorectal and gastric cancers have assessed the safety and preliminary efficacy of DNMTi or HDACi combined with PD-1/PD-L1 blockade (97).

### 3.2 Cancer vaccines and gut microbiota

Neoantigen and mRNA vaccines: Therapeutic cancer vaccines aim to stimulate the patient’s own immune system to recognize and attack tumor cells. mRNA vaccine technology, which allows rapid design and production, has emerged as a promising platform in cancer immunotherapy. mRNA vaccines can activate both innate and adaptive immunity, inducing robust humoral and cellular responses that enhance anti-tumor activity. Preclinical studies have demonstrated the potent ability of mRNA vaccines to boost immune responses and improve survival. For instance, an mRNA vaccine encoding tumor neoantigens significantly expanded functional T-cell responses, inhibited tumor growth, and improved survival in a mouse model of colorectal cancer (98). In the realm of personalized medicine, the U.S. National Cancer Institute (NCI) in collaboration with Moderna developed a personalized mRNA vaccine targeting *KRAS* mutations. In a phase I/II trial, this vaccine showed a favorable safety profile and induced mutation-specific T-cell responses in 4 patients with metastatic GI cancers (99). Another personalized mRNA vaccine, RO7198457 (BNT122) developed by BioNTech/Genentech, was tested in a phase I trial for resected pancreatic ductal adenocarcinoma. Sixteen patients received the vaccine in combination with atezolizumab (anti–PD-L1) and adjuvant chemotherapy (mFOLFIRINOX) after surgery (100). The results were encouraging: 50% of patients mounted T-cell responses against neoantigens, and these responders had significantly improved disease-free survival at three years compared to non-responders (101). This study provided proof-of-concept that personalized neoantigen vaccines can elicit meaningful immune activity in GI cancers, with potential clinical benefit.

Oncolytic viruses and peptide vaccines: Oncolytic virotherapy uses viruses that selectively infect and lyse tumor cells, releasing tumor antigens in the process and converting an “immune-cold” tumor into a “hot” one. Oncolytic viruses can thereby improve ICI responses and overcome resistance (102, 103). Preclinical models have shown that engineered oncolytic viruses can target cancer stem cells and synergize with T-cell therapies to suppress growth of therapy-resistant tumors (104, 105). Moreover, oncolytic viruses can be armed to express immunostimulatory genes or combined with vaccines to further boost anti-tumor immunity (106). Several phase I/II trials in gastric and colorectal cancer have evaluated oncolytic viruses (e.g. HSV-1 or adenovirus vectors) together with peptide vaccines. For example, HER-Vaxx (a peptide vaccine targeting HER2) has been tested with an oncolytic virus (107), as have vaccines against other antigens like LY6K (108) and the neoantigen ensemble OTSGC-A24 (109). These studies have demonstrated that the combinations are safe and well-tolerated in patients, with a subset of patients showing tumor stability or regression. In microsatellite-stable colorectal cancer, a neoantigen vaccine combined with an oncolytic virus similarly showed some patients achieving disease control (110, 111). While the objective response rates are modest so far, these trials suggest that multi-modal immunotherapy (virotherapy + vaccination + ICIs) is a feasible strategy to provoke immune responses even in resistant GI tumors. Ongoing studies will determine if such combinations can significantly extend progression free or overall survival.

Gut microbiota modulation: The gut microbiome has emerged as a key regulator of systemic immunity and can profoundly affect responses to chemotherapy and immunotherapy in GI cancers (112). There is mounting evidence from preclinical models and clinical correlative studies that manipulating the gut microbiota can alter tumor drug sensitivity (113). In mouse models carrying human GI tumor xenografts, treatment with broad-spectrum antibiotics significantly attenuated the anti-tumor efficacy of oxaliplatin chemotherapy and CpG-ODN immunotherapy, indicating that certain commensal bacteria enhance treatment responses (114). These findings imply that an intact, favorable microbiome is required for optimal therapy effect, possibly by promoting an immunostimulatory environment. Fecal microbiota transplantation (FMT) from immunotherapy responders is being investigated to overcome immunotherapy resistance. In an exploratory trial involving patients with anti PD-1 refractory GI cancers (including gastric, esophageal, and liver cancers), administering FMT from a donor (followed by anti PD-1 rechallenge) achieved an objective response rate of ~20% and a disease control rate of 95% (115, 116). This suggests that introducing a “favorable” microbiome can restore responsiveness in a subset of resistant patients. Additionally, specific bacterial taxes have been linked to better immunotherapy outcomes. For example, Han et al. (117) found that gastric cancer patients with higher relative abundance of *Lactobacillus* in their gut had improved responses to ICIs and longer progression-free survival (117). This raises the intriguing possibility of using certain probiotics as adjuvants to immunotherapy. Ongoing trials are examining probiotic supplementation or diet modifications in combination with ICIs in GI malignancies (118). While the field is still nascent, harnessing the gut microbiota holds promise for modulating the TIME and combating resistance.

Engineered cell therapies: Adoptive cell therapies have revolutionized the treatment of hematologic malignancies, and efforts are underway to translate this success to solid tumors including GI cancers. However, solid tumors pose unique hurdles such as heterogenous antigen expression, an immunosuppressive TIME, and physical barriers to T-cell infiltration. Tumors can develop resistance to engineered T cells through various mechanisms: acquisition of cancer stem cell–like properties, antigen loss or downregulation, upregulation of inhibitory molecules, and increased infiltration of suppressive cells. To address these challenges, new generations of CAR-T cells are being designed with enhanced functions. For example, researchers have created CAR-T cells that co-express a secretable PD-1–TREM2 bispecific antibody fragment, allowing the CAR-T cells to locally block inhibitory signals in the TME as they engage tumor cells (119). CAR-NK cells are also being explored; Torchiaro et al. (120) demonstrated that CAR-NK cells targeting the antigen mesothelin significantly suppressed tumor growth in a resistant colorectal cancer model (120). Combinatorial approaches such as CAR-T cells given alongside dendritic cell vaccines targeting cancer stem cell antigens, or CAR-T combined with checkpoint blockade, have yielded superior tumor regression and formation of memory T cells *in vivo* (121). There is interest in “armored” CAR-T cells that secrete cytokines or checkpoint blockers to modify the TME, as well as multi-target CARs that can recognize several antigens to mitigate immune escape.

Traditional CAR-T cells are based on αβ T cells, which require MHC presentation of antigen. An emerging strategy is to use γδ T cells, which are MHC-unrestricted and have inherent tropism to tissue sites (122). CAR-engineered γδ T cells have been shown to better penetrate solid tumors and resist some immunosuppressive factors (123, 124). Early studies indicated that CAR-γδ T cells can bypass MHC-related evasion and maintain activity in hypoxic, adenosine-rich tumor areas. Currently, there are two ongoing clinical trials investigating CAR-γδ T cells in hepatocellular carcinoma (NCT06364787, NCT06364800). Additionally, T cell receptor–modified T cells (TCR-T) targeting intracellular cancer antigens have entered trials (125, 126). Kim SP et al. (127) tested TCR-T cells specific to common GI tumor antigens and found they could mediate tumor regression in a subset of patients (NCT01174121, NCT03412877) (127). For example, TCR-T cells recognizing KRAS G12D mutations are being evaluated in phase I/II studies (NCT05194735). Tumor-infiltrating lymphocyte (TIL) therapy, where autologous TILs are expanded ex vivo and reinfused, has shown success in melanoma and is now being applied to GI cancers (128). A recent study using a refined approach (selecting tumor-reactive TILs and combining PD-1 blockade) achieved significant tumor shrinkage in ~24% of heavily pretreated metastatic GI cancer patients (129). This suggests that even in GI tumors considered “cold,” there exist T cells capable of mediating rejection if appropriately activated and unleashed.

Looking ahead, an increasing number of novel immunotherapeutic drugs and strategies are being reported as promising approaches to overcome tumor immune tolerance. However, diverse resistance mechanisms and high tumor heterogeneity remain significant challenges. To address these challenges, strategies such as multi-target combination therapies, modulation of the tumor microenvironment, and identification of predictive biomarkers are required to enhance therapeutic efficacy and delay resistance. The latest ongoing clinical trials are summarized in the table below.

**Table 1** provides an overview of select clinical trials testing these novel immunotherapeutic approaches in GI cancers, highlighting the landscape of multi-modal strategies aimed at overcoming TIME-mediated resistance.

**Table 1:** Clinical trials of novel immunotherapies for gastrointestinal tumors in the recruitment stage.

Therapy type	Registration number	Study phase	Intervention/Treatment	Cancer models
mRNA Vaccine	NCT05227378	Not Applicable	Neoantigen tumor vaccine with or without PD-1/L1	Gastric Cancer
	NCT05192460	Not Applicable	Neoantigen tumor vaccine with or without PD-1/L1	Gastric Cancer, Esophageal Cancer, Liver Cancer
	NCT06019702	I	iNeo-Vac-R01	Digestive System Neoplasms
	NCT06026800	I	iNeo-Vac-R01	Digestive System Neoplasms
	NCT06026774	I	iNeo-Vac-R01 in combination with standard adjuvant therapy	Digestive System Neoplasms
	NCT03468244	Not Applicable	Personalized mRNA Tumor Vaccine	Advanced Esophageal Squamous Carcinoma, Gastric-Adenocarcinoma, Pancreatic Adenocarcinoma, Colorectal-Adenocarcinoma
DC cell vaccine	NCT01885702	I	DC vaccination	Colorectal Cancer
	NCT04708470	I	Bintrafusp Alfa, PDS01ADC, Entinostat	Oropharyngeal Cancer, Neck Cancer, Human-Papillomavirus, HPV, Anal Cancer, Cervical Cancer, Penile Cancer, Vulvar Cancer, Vaginal Cancer, Colon-Cancer
	NCT06751953	Not Applicable	Conventional third-line therapy, Neoantigen-loaded DC vaccine	Colorectal Cancer (CRC)
	NCT06751940	Not Applicable	Combination Product: Conventional second-line therapy, Neoantigen-loaded DC vaccine	Colorectal Cancer (CRC)
	NCT06522919	II	Autologous Dendritic Cell (DC) Vaccine	Colorectal Cancer Metastatic, Microsatellite Stable-Colorectal Carcinoma, Refractory Mismatch-repair-proficient (pMMR) Metastatic Colorectal Cancer
	NCT06545630	I	Tumor antigen-sensitized DC vaccine	Colorectal Cancer
	NCT03410732	II	Activated DCs, Radical surgery only	Gastric Cancer
	NCT02632201	I	PIK-HER2, DC-PMAT	Liver Metastasis, Gastric Cancer
Oncolytic virus	NCT05733611	II	RP2, RP3, Atezolizumab, Bevacizumab	Refractory Metastatic Colorectal Cancer, pMMR, MSS
	NCT06283303	I	T3011 hepatic artery infusion, Toripalimab, Regorafenib	Colorectal Cancer Metastatic
	NCT06283134	I	BioTTT001 hepatic artery infusion, Toripalimab, Regorafenib	Colorectal Cancer Metastatic
	NCT05860374	Early I	Recombinant oncolytic herpes simplex virus type 1 (R130)	Sarcoma, Carcinoma, Breast Cancer, Pancreatic Cancer. Colorectal Cancer, Gastric Cancer, Liver Cancer, Lung-Cancer, Gynecologic Cancer
	NCT05427487	I	IVX037, sintilimab	Colorectal Cancer, Gastric Cancer, Ovarian Cancer
	NCT06265025	I	GM103 (Part A), GM103 (Part B), GM103 and Pembrolizumab (Part C)	Head and Neck Cancer, Malignant Melanoma, Colorectal-Cancer, Renal Cell Carcinoma, Cervical Cancer, Breast-Cancer
	NCT06444815	I	VET3-TGI, Pembrolizumab	Solid Tumor, Microsatellite Stable Colorectal Cancer Head and Neck Squamous Cell Carcinoma, Cervical-Cancer, Kidney Cancer, Renal Cell Carcinoma, Melanoma -Stage IV, Merkel Cell Carcinoma of Skin Mesothelioma, Non-small Cell Lung Cancer
	NCT03740256	I	CAdVEC	Bladder Cancer, Head and Neck Squamous Cell Carcinoma, Cancer of the Salivary Gland, Lung Cancer Breast Cancer, Gastric Cancer, Esophageal Cancer Colorectal Cancer, Pancreatic Adenocarcinoma, Solid-Tumor
	NCT05886075	Early I	Recombinant oncolytic herpes simplex virus type 1 (R130)	Lung Cancer, Bronchial Cancer, Non-Small Cell Lung-Cancer, Small Cell Lung Cancer, Sarcoma, Colorectal-Cancer, Gastric Cancer, Liver Cancer, Breast Cancer Pancreatic Cancer, Head and Neck Cancer, Ovarian Cancer
	NCT06283121	II	BioTTT001, SOX regimen, Toripalimab	Gastric Cancer, Metastatic
	NCT05860374	Early I	Recombinant oncolytic herpes simplex virus type 1 (R130)	Sarcoma, Carcinoma, Breast Cancer, Pancreatic Cancer Colorectal Cancer, Gastric Cancer, Liver Cancer, Lung-Cancer, Gynecologic Cancer
	NCT05427487	I	IVX037, Sintilimab	Colorectal Cancer, Gastric Cancer, Ovarian Cancer
	NCT06508307	I	Oncolytic Vaccinia Virus GC001	Sarcoma, Cervical Cancer, Colon Cancer, Lung Cancer Ovarian Cancer, Pancreatic Cancer, Hepatocellular, Carcinoma, Breast Cancer, Gastric Cancer
	NCT03740256	I	CAdVEC	Bladder Cancer, Head and Neck Squamous Cell Carcinoma Cancer of the Salivary Gland, Lung Cancer, Breast Cancer Gastric Cancer, Esophageal Cancer, Colorectal Cancer Pancreatic Adenocarcinoma, Solid Tumor
	NCT05886075	Early I	Recombinant oncolytic herpes simplex virus type 1 (R130)	Lung Cancer, Bronchial Cancer, Non-Small Cell Lung-Cancer, Small Cell Lung Cancer, Sarcoma, Colorectal-Cancer, Gastric Cancer, Liver Cancer, Breast Cancer Pancreatic Cancer, Head and Neck Cancer, Ovarian Cancer
CAR-NK Cell	NCT05213195	I	NKG2D CAR-NK	Refractory Metastatic Colorectal Cancer
	NCT06464965	I	CB CAR-NK182	Gastric Cancer, Pancreas Adenocarcinoma
	NCT06358430	I	Fludarabine Phosphate, Cyclophosphamide, Cetuximab TROP2-CAR-NK Cells, Rimiducid (AP1903), Lymphodepleting Chemotherapy	Colorectal Cancer, Minimal Residual Disease
	NCT02839954	I, II	Anti-MUC1 CAR-pNK cells	Hepatocellular Carcinoma, Non-small Cell Lung Cancer Pancreatic Carcinoma, Triple-Negative Invasive Breast-Carcinoma, Malignant Glioma of Brain, Colorectal Carcinoma, Gastric Carcinoma

## 4 Conclusions

Therapeutic resistance in gastrointestinal tumors remains a key barrier to successful treatment outcomes. This review has illustrated the multifaceted roles of the tumor immune microenvironment in mediating immunotherapy resistance in GI cancers. Over the past few years, innovative treatment strategies such as combination regimens, cancer vaccines, microbiome modulation, and adoptive cell therapies have been developed to counteract resistance, and some have achieved encouraging progress, especially in targeted or microenvironment-tailored approaches. However, the heterogeneity of tumors and the diverse mechanisms of resistance demand further solutions. Future research should focus on multi-target combination therapies, overcoming tumor immune escape pathways, and identifying robust predictive biomarkers, with the goal of achieving personalized and precise therapy for GI cancer patients.

With continued multidisciplinary collaboration and rigorous clinical investigations, there is optimism that novel therapeutic strategies will provide more effective treatment options for patients with GI tumors and improve their prognosis. Overcoming immunotherapy resistance will likely require an integrated approach that remodels the TIME, attacks tumor-intrinsic resistance nodes, and harnesses the patient’s immune system in a concerted fashion. As our understanding of the TIME deepens and new technologies emerge, the prospects for converting currently unresponsive GI tumors into ones that can be durably controlled by the immune system are becoming increasingly tangible.
